# Associations between certolizumab pegol serum levels, anti-drug antibodies and treatment response in patients with inflammatory joint diseases: data from the NOR-DMARD study

**DOI:** 10.1186/s13075-019-2009-5

**Published:** 2019-11-29

**Authors:** Johanna Elin Gehin, Guro Løvik Goll, David John Warren, Silje Watterdal Syversen, Joseph Sexton, Eldri Kveine Strand, Tore Kristian Kvien, Nils Bolstad, Elisabeth Lie

**Affiliations:** 1 Department of Medical Biochemistry, Oslo University Hospital, Radiumhospitalet, Nydalen, Box 4953, 0424 Oslo, Norway; 2 Faculty of Medicine, University of Oslo, Oslo, Norway; 3 Department of Rheumatology, Diakonhjemmet Hospital, Oslo, Norway; 4 Lillehammer Hospital for Rheumatic Diseases, Lillehammer, Norway

**Keywords:** TNF-inhibitors, Certolizumab pegol, Serum drug levels, Anti-drug antibodies, Inflammatory joint diseases, Axial spondyloarthritis, Rheumatoid arthritis, Psoriatic arthritis

## Abstract

**Objectives:**

To identify a therapeutic target interval for certolizumab pegol drug levels and examine the influence of anti-drug antibodies in patients with inflammatory joint diseases.

**Methods:**

Certolizumab pegol and anti-drug antibody levels were measured in serum samples collected after 3 months of certolizumab pegol treatment in 268 patients with inflammatory joint diseases (116 axial spondyloarthritis, 91 rheumatoid arthritis and 61 psoriatic arthritis) in the NOR-DMARD study. Treatment response was defined by Ankylosing Spondylitis Disease Activity Score Clinically important improvement in axial spondyloarthritis, European League Against Rheumatism good/moderate response in rheumatoid arthritis, and improvement in 28-joint Disease Activity Score of ≥ 0.6 in PsA. Serum drug levels and anti-drug antibodies were analysed using automated in-house assays.

**Results:**

Certolizumab pegol serum levels varied considerably between individuals (median (IQR) 32.9 (17.3–43.9) mg/L). Certolizumab pegol level ≥ 20 mg/L was associated with treatment response for the total inflammatory joint disease population, with odds ratio (OR) 2.3 (95% CI 1.2–4.5, *P* = 0.01) and OR 1.9 (95% CI 1.0–3.5, *P* = 0.05) after 3 and 6 months of treatment, respectively. For individual diagnoses, this association was most consistent for axial spondyloarthritis, with OR 3.4 (95% CI 1.0–11.1, *P* < 0.05) and OR 3.3 (95% CI 1.0–10.8, *P* < 0.05), respectively. Certolizumab pegol level > 40 mg/L was not associated with any additional benefit for any of the diagnoses. Anti-drug antibodies were detected in 6.1% (19/310) of samples and were associated with low certolizumab pegol levels (*P* < 0.01).

**Conclusions:**

Serum certolizumab pegol levels 20–40 mg/L were associated with treatment response in inflammatory joint diseases. Our study is the first to show this association in axial spondyloarthritis and psoriatic arthritis patients. The results suggest a possible benefit of therapeutic drug monitoring in patients with inflammatory joint disease on certolizumab pegol treatment.

**Trial registration:**

NCT01581294, April 2012.

## Introduction

Tumour necrosis factor alpha inhibitors (TNFi), such as certolizumab pegol (CZP), have substantially improved the management of inflammatory joint diseases (IJD). However, a significant proportion of patients do not respond adequately to treatment [1–4]. Low drug levels and development of anti-drug antibodies (ADAb) have previously been shown to be associated with lack of response to TNFi [5–10].

Therapeutic drug monitoring (TDM) can help clinicians tailor treatment with biologic drugs. TDM has the potential to reduce under- and overtreatment and has been suggested to improve effectiveness, safety and cost-effectiveness of treatment with biologic drugs. For TDM to be validated as a clinical tool, therapeutic intervals must be identified. Previous reports suggest therapeutic intervals in patients treated with infliximab [5, 6, 11, 12] and adalimumab [7, 8, 13]. Measurement of serum levels has become common clinical practice in many rheumatology, gastroenterology and dermatology centres for these drugs across Europe. Knowledge on optimal serum drug levels of other TNFi, such as CZP, is largely lacking. In addition, the majority of data on serum drug concentrations in rheumatic diseases are on patients with rheumatoid arthritis (RA) only.

CZP is a PEGylated humanised Fab’ fragment of a recombinant monoclonal murine antibody against TNFα. Though CZP is extensively used in treatment of IJD, knowledge about the optimal serum drug level is limited. An association between CZP levels and treatment response has previously been shown in a prospective observational study of patients with RA [10].

It is well known that a considerable proportion of patients develop ADAb to infliximab and adalimumab, often leading to low drug levels and treatment failure [8, 9, 14–17]. Knowledge about the incidence and clinical relevance of ADAb to CZP is very limited. Jani et al. detected ADAb in 37% of RA patients, and the presence of ADAb was significantly associated with lower drug levels, but not with clinical outcomes [10].

The main objective of our study was to examine the association between serum CZP levels and treatment response in order to identify a therapeutic target interval in patients with IJD. In addition, we wanted to assess the frequency and clinical relevance of early ADAb development in patients treated with CZP.

## Methods

### The NOR-DMARD registry and patient selection

The NOR-DMARD registry is a longitudinal observational study including adult patients with IJD starting treatment with biologic disease-modifying antirheumatic drugs (bDMARDs) [18]. Biobank samples in the NOR-DMARD study are collected at baseline and at the 3-month follow-up visit. Clinical assessments are performed at baseline, 3, 6, 9 and 12 months and every 6 months thereafter.

In the current study, we included NOR-DMARD patients enrolled in the registry from January 2013 to December 2016 with a clinical diagnosis of axial spondyloarthritis (axSpA) (*n* = 116), RA (*n* = 91), psoriatic arthritis (PsA) (*n* = 61) and other IJD (*n* = 42) starting treatment with CZP and with available biobank samples at the 3-month follow-up visit. Serum samples were non-trough, collected at the 3-month visit and stored in a biobank freezer at − 80 °C. Clinical data from baseline, 3- and 6-month follow-up visits were used.

Patients with axSpA, RA and PsA with available baseline disease activity score were included in response analyses (*n* = 245; 110 axSpA, 81 RA, 54 PsA). Complete data for the 6-month follow-up visit were available in 60% (150/252) of patients. An additional 38% (95/252) became eligible for the 6-month response analyses when carrying forward their 3-month values. For missing 3-month data (*n* = 24), values from 6 months were carried backwards. For patients with available data on some of the composite score values (*n* = 3 at 3 months and *n* = 3 at 6 months), only the missing components were imputed. For those with all components missing, the disease activity scores themselves were imputed (*n* = 24 at 3 months and *n* = 92 at 6 months). Seven patients were excluded from analyses due to inadequate 3- and 6-month data and unknown reason for discontinuation. One patient had non-response imputed because of discontinuation due to lack of efficacy before the 3-month visit.

### Clinical response

Clinical data for the analyses were collected at the 3- and 6-month follow-up visit. Disease activity was assessed by Ankylosing Spondylitis Disease Activity Score-C-reactive protein (ASDAS-CRP) for axSpA [19] and 28-joint Disease Activity Score-erythrocyte sedimentation rate (DAS28-ESR) [20] for RA and PsA. Treatment response was defined by ASDAS Clinically important improvement (CII) (defined by a reduction of ≥ 1.1 units in ASDAS-CRP) in axSpA [19], European League Against Rheumatism (EULAR) good/moderate response in RA [21] and DAS28 improvement ≥ 0.6 in PsA [22]. Patients with other IJD were not included in response analyses.

### Adverse events

Adverse events during the first 700 days of treatment, where a relation to CZP treatment was suspected, were included in analyses. Adverse event severity was classified according to MedDRA [23].

### Measurement of CZP levels and ADAb

CZP levels were analysed retrospectively in non-trough serum samples collected at 3 months using an in-house, European In-Vitro Diagnostic Devices Directive-compliant, time-resolved fluorometric assay automated on the AutoDELFIA (PerkinElmer, Waltham, MA, USA) immunoassay platform. The assay uses human recombinant TNFα as capture reagent. Active drug binding to the TNFα solid phase is detected using a europium-labelled anti-kappa light chain monoclonal antibody [24]. ADAb was detected by a principal assay measuring neutralising ADAb and two confirmational assays. The principal assay is based on the ability of ADAb to inhibit binding of europium-labelled drug to a TNFα solid phase [24]. The confirmational tests were an antigen-bridging test and a 3-step fluorometric assay. Results were defined as positive if the principal assay and at least one confirmational assay were positive.

### Statistical analyses

For differences in baseline demographics and clinical variables between groups, independent samples *t*-test, Mann-Whitney *U* test or *χ*^2^ tests were used, as appropriate. Statistical tests were two-sided with level of significance set at 0.05. Associations between CZP levels and improvement in disease activity score and response were assessed by multivariate linear and logistic regression (adjusting for age, sex and prior bDMARD use (yes/no)), respectively. Statistical analyses were performed using IBM SPSS Statistics, Version 25.

## Results

### Baseline characteristics

Baseline characteristics are shown in Table 1, stratified by low (< 20 mg/L) vs. high (≥ 20 mg/L) CZP level at 3 months. Female gender was associated with CZP level ≥ 20 mg/L in RA patients. No significant differences between groups were found for other demographic or clinical data.

**Table 1:** Comparison of baseline characteristics across patients with low (< 20 mg/L) vs. high (≥ 20 mg/L) CZP serum level

Axial spondyloarthritis	All	CZP low (< 20 mg/L)	CZP high (≥ 20 mg/L)	*P* value
	(*n* = 116)	(*n* = 26)	(*n* = 90)	
Age, years, mean (SD)	42 (12)	43 (11)	41 (12)	0.61
Female, *n* (%)	54 (47)	14 (54)	40 (44)	0.40
Disease duration, years, median (IQR)*	2.6 (0.6–14.1)	3.6 (1.7–11.7)	2.3 (0.3–14.8)	0.39
ASDAS-CRP, mean (SD)	2.6 (1.0)	2.4 (0.9)	2.7 (1.0)	0.28
HLA-B27 positive, *n* (%)	87 (75)	17 (65)	70 (81)	0.09
Prior use of biologic DMARD, *n* (%)	39 (34)	10 (40)	29 (33)	0.54
Concomitant conventional synthetic DMARD, *n* (%)	22 (19)	2 (8)	20 (22)	0.10
Rheumatoid arthritis	All	CZP low (< 20 mg/L)	CZP high (≥ 20 mg/L)	*P* value
	(*n* = 91)	(*n* = 23)	(*n* = 68)	
Age, years, mean (SD)	54 (14)	54 (16)	54 (14)	0.90
Female, *n* (%)	72 (79)	13 (57)	59 (87)	< 0.05
Disease duration, years, median (IQR)**	10.1 (2.1–18.9)	17.4 (6.8–23.5)	7.4 (2.0–14.9)	0.10
DAS28, mean (SD)	4.0 (1.4)	3.5 (1.1)	4.2 (1.5)	0.08
RF-positive, *n* (%)	55 (61)	12 (52)	43 (66)	0.23
Anti-CCP positive, *n* (%)	59 (66)	13 (57)	46 (71)	0.21
Prior use of biologic DMARD, *n* (%)	44 (48)	14 (64)	30 (45)	0.13
Concomitant conventional synthetic DMARD, *n* (%)	67 (74)	16 (70)	51 (75)	0.53
Psoriatic arthritis	All	CZP low (< 20 mg/L)	CZP high (≥ 20 mg/L)	*P* value
	(*n* = 61)	(*n* = 17)	(*n* = 44)	
Age, years, mean (SD)	50 (11)	48 (12)	51 (11)	0.45
Female, *n* (%)	40 (66)	12 (71)	28 (64)	0.61
Disease duration, years, median (IQR)***	6.6 (1.5–13.2)	5.4 (1.3–13.5)	6.9 (1.6–13.2)	0.76
DAS28, mean (SD)	3.9 (1.3)	3.9 (1.8)	3.9 (1.2)	0.99
Prior use of biologic DMARD, *n* (%)	30 (49)	10 (59)	20 (47)	0.39
Concomitant conventional synthetic DMARD, *n* (%)	38 (67)	8 (53)	30 (71)	0.20

Data available in *n* = *68, **68, ***40 patients

*CZP* certolizumab pegol, *ASDAS-CRP*, Ankylosing Spondylitis Disease Activity Score-C-reactive protein, *DAS28* 28-joint Disease Activity Score, *RF* rheumatoid factor, *Anti-CCP* anti-cyclic citrullinated peptides, *DMARD* disease-modifying antirheumatic drug, *SD* standard deviation, *IQR* interquartile range

### Distribution of CZP serum levels

**Fig. 1:**
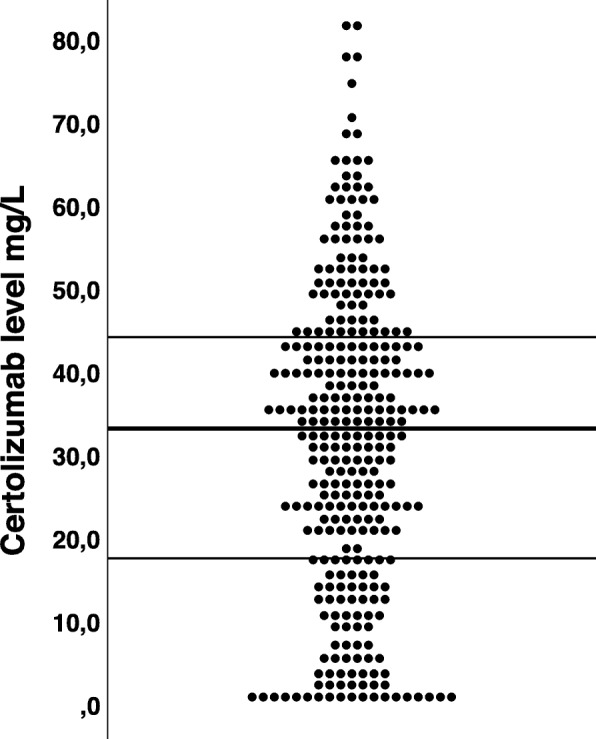
Distribution of certolizumab serum levels (total inflammatory joint disease population) at 3 months, mg/L. Median (IQR) 32.9 (17.3–43.9)

CZP serum levels 3 months after treatment initiation showed considerable variation between individuals (Fig. 1). For the total IJD population, median (interquartile range (IQR)) CZP level was 32.9 (17.3–43.9) mg/L. Stratified by diagnosis, median (IQR) CZP level was 35.0 (21.3–45.3) mg/L in axSpA patients, 34.7 (17.6–44.6) mg/L in RA and 31.0 (13.6–39.9) mg/L in PsA. In the total population, 17 patients (5.5%) had CZP levels < 1 mg/L, 30 patients (9.7%) had serum levels 1–9.9 mg/L, 35 (11.3%) 10–19.9 mg/L, 55 (17.7%) 20–29.9 mg/L, 71 (22.9%) 30–39.9 mg/L and 102 (32.9%) ≥ 40 mg/L. Data for the administered dose of CZP were available in 95% of patients at 3 months. The majority of patients, 85%, were on standard dose, 200 mg every second week at 3 months. Among patients who were not on standard dose, 24 received 200 mg with a longer dosing interval, 17 received a higher dose (either by shorter interval between injections or higher dose) and 1 patient had discontinued treatment before 3 months. All patients were given the standard loading dose of 400 mg at weeks 0, 2 and 4.

### Association between CZP levels and treatment response

**Fig. 2:**
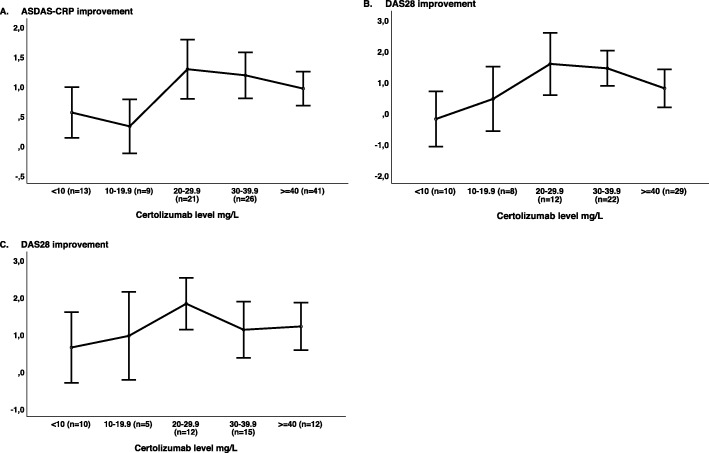
Improvement in disease activity from baseline (unadjusted means (95% CI)) at 3 months by certolizumab pegol level: **a** ASDAS-CRP improvement in axial spondyloarthritis. **b** DAS28 improvement in rheumatoid arthritis. **c** DAS28 improvement in psoriatic arthritis. ASDAS-CRP, Ankylosing Spondylitis Disease Activity Score-C-reactive protein; DAS28, 28-joint Disease Activity Score

In order to identify thresholds for drug level concentration-effect curves after 3 months of treatment were made for axSpA, RA and PsA patients (Fig. 2a–c). For all three diagnoses, the curves illustrate that patients with CZP level 20–39.9 mg/L had the largest mean improvement in disease activity from baseline. In the multivariate analysis, a serum CZP level ≥ 20 mg/L was associated with ASDAS improvement at 3 months (*β* = 0.6, (95% confidence interval (CI) 0.1–2.0), *P* = 0.01) and 6 months (*β* = 0.6, (95% CI 0.2–1.1), *P* < 0.01) in axSpA patients. In RA patients, serum CZP level ≥ 20 mg/L was associated with greater improvement in DAS28 at 3 months (*β* = 0.9 (95% CI 0.0–1.7), *P* = 0.04). Further, the association between CZP level ≥ 20 mg/L and DAS28 improvement at 6 months was borderline significant (*β* = 0.8 (95% CI − 0.1–1.8), *P* = 0.08). In PsA patients, there was a trend for CZP level ≥ 20 mg/L to be associated with greater improvement in DAS28 at 3 and 6 months; however, it did not reach statistical significance (*β* = 0.5 (95% CI − 0.2–1.3), *P* = 0.14, and *β* = 0.4 (95% CI − 0.3–1.1), *P* = 0.28).

**Fig. 3:**
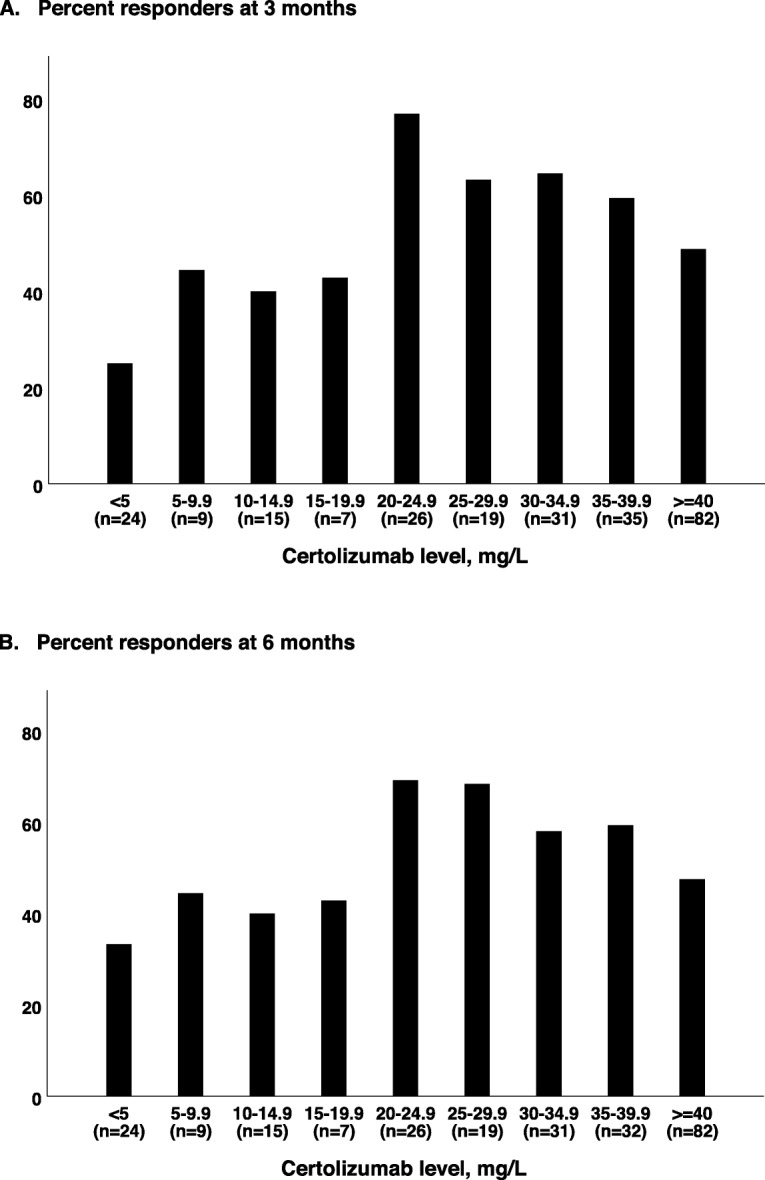
Proportion of responders (total inflammatory joint disease population) at **a** 3 months and **b** 6 months, stratified by certolizumab level (mg/L) at 3 months. Response in axial spondyloarthritis was defined by Clinically important improvement the Ankylosing Spondylitis Disease Activity Score, in rheumatoid arthritis as European League Against Rheumatism good/moderate response, and in psoriatic arthritis as improvement of ≥ 0.6 in 28-joint Disease Activity Score

**Fig. 4:**
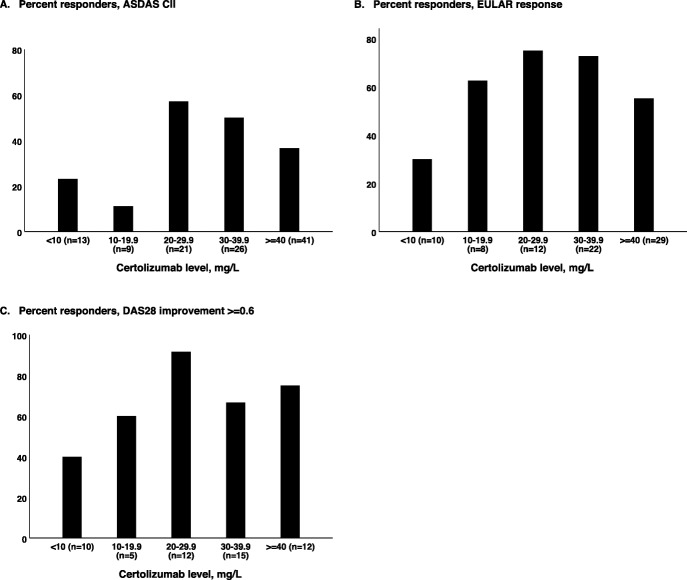
Proportion of responders at 3 months, stratified by certolizumab level (mg/L). **a** ASDAS CII responders in axial spondyloarthritis. **b** EULAR good/moderate response in rheumatoid arthritis. **c** DAS28 improvement ≥ 0.6 in psoriatic arthritis. ASDAS CII, Clinically important improvement the Ankylosing Spondylitis Disease Activity Score; EULAR good/moderate, European League Against Rheumatism good/moderate response; DAS28 improvement ≥ 0.6, improvement of ≥ 0.6 in 28-joint Disease Activity Score

Furthermore, the associations between serum drug levels and response to treatment, defined as ASDAS CII in axSpA, EULAR good/moderate response in RA, and DAS28 improvement ≥ 0.6 in PsA were examined. The proportions of responders after 3 and 6 months, for the total IJD population and for axSpA, RA and PsA separately, stratified by the CZP serum level at 3 months, are shown in Table 2 and Fig. 3a, b and Fig. 4a–c. Odds ratio (OR) (95% CI) for response in patients with CZP level ≥ 20 mg/L, versus < 20 mg/L, after 3 and 6 months of treatment are also shown in Table 2. Having a serum CZP level ≥ 20 mg/L was associated with response at 3 and 6 months for all three diagnoses combined (OR 2.3 (95% CI 1.2–4.5, *P* = 0.01), OR 1.9 (95% CI 1.0–3.5, *P* = 0.05), respectively). However, CZP levels ≥ 40 mg/L were not associated with any additional benefit, and response rates were, on the contrary, lower across all diagnoses.

**Table 2:** Response*(%) at 3 and 6 months, stratified by certolizumab pegol level at 3 months

	Overall	CZP < 20 mg/L	CZP 20–39.9 mg/L	CZP ≥ 40 mg/L	OR** (95% CI)	P**
	Responders* after 3 months					
All patients (*n* = 245)	53%	35%	65%	49%	2.3 (1.2–4.5)	< 0.05
axSpA (*n* = 110)	40%	18%	53%	37%	3.4 (1.0–11.1)	< 0.05
RA (*n* = 81)	61%	44%	74%	55%	1.5 (0.5–5.1)	0.48
PsA (*n* = 54)	69%	47%	77%	77%	4.3 (1.0–17.9)	< 0.05
	Responders* after 6 months					
All patients (*n* = 245)	52%	38%	63%	48%	1.9 (1.0–3.5)	0.05
axSpA (*n* = 110)	40%	18%	55%	34%	3.3 (1.0–10.8)	< 0.05
RA (*n* = 81)	57%	50%	62%	55%	1.1 (0.3–3.4)	0.92
PsA (*n* = 54)	70%	53%	77%	70%	3.3 (0.8–13.3)	0.09

*CZP* certolizumab pegol, *OR* odds ratio, *CI* confidence interval

*Response in axial spondyloarthritis (axSpA) was defined by clinically important improvement the Ankylosing Spondylitis Disease Activity Score, in rheumatoid arthritis (RA) as European League Against Rheumatism good/moderate response, and in psoriatic arthritis (PsA) as improvement of ≥ 0.6 in 28-joint Disease Activity Score

**Multivariate logistic regression comparing response in patients with CZP < 20 vs ≥ 20 mg/L, adjusting for age, sex and prior biologic disease-modifying antirheumatic drug use (yes/no)

### Association between CZP levels and adverse events

In the total IJD population, 69 patients had registered one or more infections (the majority had one (*n* = 42) or two (*n* = 20) infections). All infections were mild or moderate in severity.

With patients stratified by CZP levels < 20, 20–39.9 and ≥ 40 mg/L, the proportions of patients who had ≥ 1 infection were 21%, 22% and 24%, respectively, and the proportions of patients who had ≥ 2 infections were 7%, 8% and 11%, respectively. While there is a slight trend towards more infections with higher drug levels, the differences were not statistically significant. Only three patients had ≥ 5 infections, and all these patients had CZP levels ≥ 40 mg/L.

A total of 111 patients had one or more “other” adverse events, most commonly one (*n* = 74) or two (*n* = 28). There was one severe allergic skin reaction, while the remaining adverse events were mild or moderate in severity. The proportion of patients with ≥ 1 and ≥ 2 “other” adverse events did not differ between groups stratified by CZP levels.

### Frequency and clinical significance of ADAb at 3 months sampling

After 3 months of treatment, 19 of 310 (6.1%) patients were ADAb positive (6 axSpA, 5 RA, 4 PsA and 4 other IJD). ADAb-positive patients had significantly lower CZP levels than ADAb-negative patients, i.e., median (IQR) 1.0 (0.2–6.8) vs. 34.4 (21.2–44.7) mg/L (*P* < 0.01). ADAb was detected in 53% (9/17) of those with CZP < 1 mg/L, compared to 3% (10/293) of those with CZP ≥1 mg/L.

Response data were available for 245 patients. Of these, only 1 out of 11 (9%) ADAb-positive patients was classified as a responder at 3 months. Among ADAb-negative patients with response data, 129/234 (55%) were responders.

Among RA patients, 4 of 5 (80%) ADAb-positive patients used concomitant synthetic DMARDs (mostly methotrexate). Numbers for ADAb-negative patients were 63 out of 86 (73%).

Eight patients experienced one or more injection-site reaction, and all of these were ADAb negative at 3 months.

## Discussion

Our study is, to our knowledge, the first to demonstrate a concentration-effect curve in CZP-treated patients with axSpA and PsA. Furthermore, we confirm the concentration-effect relationship previously demonstrated in RA [10].

Considerable inter-individual variation in serum CZP concentrations on the same standard dose was revealed, suggesting both over- and undertreatment.

The association between CZP serum levels and clinical response was most consistent for patients with axSpA. Associations between serum drug levels, ADAb and clinical effect have been found in patients with ankylosing spondylitis (AS) for other TNFi [14, 25–27]; however, it has been difficult to establish a clear therapeutic target interval in this patient group [28, 29]. The high number of patients with axSpA and their relatively short median disease duration compared to other studies are among the strengths of our study. In PsA patients, data describing associations between serum level of TNFi and response are currently limited, and this study is the first to suggest a therapeutic target level for CZP in PsA. In RA patients, data showing a concentration-effect relationship for CZP have previously been published by Jani et al. [10]. In our study, the association between CZP level and DAS28 improvement was statistically significant in RA patients, while the association between CZP level and EULAR response was not. This discrepancy might be due to lack of statistical power to show an association to a dichotomous response measure, in addition to the large group of RA patients with CZP levels ≥ 40 mg/L, in which the proportion of responders was relatively low.

The magnitude of *p* values obtained in the statistical analyses is related to the number of patients with the individual diagnoses in this study. We believe this variation can likely be explained by the lower number of patients with RA and PsA compared to axSpA, rather than a true difference between the diagnoses. Our study was not powered to study individual diagnoses, but we considered it relevant to examine whether there were obvious differences between individual diagnoses.

We aimed to identify cut-off values applicable to clinical use. The thresholds for drug levels were based on explorative concentration-effect analyses (depicted in Figs. 2, 3 and 4) and were further evaluated by regression analyses. The suggested target of 20 mg/L is comparable to previous results from a study of RA patients [10] and reports in Crohn’s patients, where CZP levels ≥ 23.3 mg/L and ≥ 14.8 at weeks 10 and 12, respectively, have been shown to be associated with better outcomes [30, 31]. However, results in Crohn’s patients may not be comparable to results in patients with rheumatic diagnoses, as intestinal loss of serum proteins (including antibody-based drugs) during periods of high disease activity is a major confounder in inflammatory bowel diseases.

We were not able to identify statistically significant associations between CZP levels and adverse events (including infections). However, a high number of infections (> 5) were only seen in patients with high CZP levels. A previous study on RA patients, including but not specifically assessing CZP, has demonstrated an association between high serum levels of biologic drugs and risk of infection [32]. In our study, the number of reported injection-site reactions was too small to conclude regarding an association with ADAb.

Disease activity measures and response criteria are defined differently in axSpA, RA and PsA, which could affect interpretation and comparability of results. Due to lack of more extensive joint counts and PsA-specific measures, we measured disease activity and response by DAS28 in PsA patients for this study. Sensitivity analyses using a modified Disease Activity index for Psoriatic Arthritis, using 28 and 32 swollen/tender joint counts (DAPSA28 and DAPSA32) [33–35], were performed in PsA (data not shown). These analyses showed the same trends as analyses using DAS28, though the levels of significance declined. We believe this is a consequence of the lower proportion of respondents among PsA patients, especially in the ≥ 40 mg/L group, when using DAPSA28/-32 improvement ≥ 50% as a response measure, compared to DAS28 response. Results were similar when using DAPSA28 compared to DAPSA32. PsA is a heterogeneous disease with diverse manifestations, making disease activity measures a challenging and controversial issue. However, DAS28 is frequently used in PsA, and DAPSA28 has been shown to be useful in the absence of more extensive joint counts [33]. Results from this cohort are quite similar across diagnoses. We therefore believe it is possible to suggest a common therapeutic target level.

Serum drug levels in the present study are non-trough. Measurement of trough levels is inconvenient in clinical practice for CZP and other TNFi that are self-administered. Hence, it is useful to identify a therapeutic range for non-trough serum samples. Previous data indicate that pharmacologic testing with non-trough levels is clinically useful in TNFi-treated patients [10, 36]. In addition, it has been suggested by pharmacokinetic simulation that intra-individual variation between injections is quite small for subcutaneously dosed TNFi, i.e., adalimumab and etanercept, in RA patients [37]. Adalimumab levels have also been shown to be similar in samples from different time points during an injection cycle in Crohn’s disease [38].

A substantial number of patients had CZP levels above 40 mg/L, and the proportion of responders was lower in the ≥ 40 mg/L group than in the 20–39.9 mg/L group for all three diagnoses. While this finding might be counterintuitive, it is in agreement with what has previously been shown for DAS28 improvement in RA patients [10]. Non-responders with high CZP levels might represent a subset of patients with a low TNFα load in their disease, leading to reduced binding of CZP and large amounts of unbound drug. As with most assays used for detection of biologic drugs, our assay only measures active drug still able to bind its target. These patients could most likely receive lower dosing without increasing the risk of disease worsening, which would reduce costs and potentially also the risk of side effects. More importantly, these patients might benefit from switching to a biologic drug with another mode of action. Garcês et al. have shown that non-responders to a TNFi in the presence of detectable serum drug trough levels and no detectable ADAb had higher probability of achieving response by switching to a drug with different mode of action, rather than to another TNFi [39]. As a result of the relatively short time from patients receiving the standard loading dose to the collection of biobank samples in our study, some patients might not have reached steady-state drug levels. However, we do not believe this is a major contributor when biobank samples were collected after 3 months of treatment.

In total, ADAb against CZP were detected in 6.1% of patients after 3 months of treatment. We found that early development of ADAb was associated with low drug levels and reduced treatment response, albeit the number of ADAb-positive patients was relatively small. Our data are not able to demonstrate a protective effect of concomitant synthetic DMARDs on ADAb formation in RA patients, but the number of ADAb-positive RA patients was too small to conclude. Phase III-IV studies have shown a similar frequency (5.0–8.1%) of ADAb in RA patients [1, 2, 40, 41]. Jani et al. found ADAb in 37% of patients in non-trough serum samples in RA patients by 12 months using a sensitive radioimmunoassay [10]. Studies assessing ADAb frequency are not necessarily comparable, because of differences in patient selection, study design and methods used to measure ADAb. For this study, we chose a confirmational strategy for ADAb detection because knowledge is limited about the immunogenicity of PEGylated Fab’ fragments. Further, high concentrations of CZP in samples may interfere in ADAb assays. Admittedly, the frequency of ADAb in our study could be underestimated because ADAb was measured in non-trough samples. A possible reason for a very low drug level (< 1 mg/L) in the absence of ADAb, which was found in 8 patients, could be lack of compliance.

We were able to identify a common therapeutic target interval for CZP across patients with different IJD. Establishment of a therapeutic target interval is necessary for further validation of TDM as a clinical tool to improve efficacy of treatment with CZP. The consistent association between serum level and effect supports a benefit of personalised dosing by TDM in patients on CZP treatment, as do the considerable variability in serum levels among patients on standard dose CZP, indicating both over- and undertreatment. Biobank samples were collected at the first visit following treatment initiation, which we believe is a strength of this study. Tools to aid treatment decisions shortly after initiation of treatment are needed when using the treat-to-target strategy recommended by EULAR and EULAR-ASAS in early disease management [42–44]. The lack of data on body weight and of more extensive joint counts in PsA patients (discussed above) is a limitation of our study. These parameters are not recorded in the NOR-DMARD registry.

In conclusion, our results demonstrate that CZP serum levels vary considerably between patients with IJD on standard dose. Serum levels ≥ 20 mg/L were associated with treatment response. However, having CZP level > 40 mg/L was not associated with any additional benefit. Results were comparable between diagnoses. ADAb against CZP were associated with low drug levels and reduced treatment response. These results suggest that a therapeutic interval of 20–40 mg/L can be implemented in clinical practice for non-trough serum samples in patients with IJD, but the clinical significance of tailoring TNFi treatment in IJD by TDM should be further explored in randomised controlled clinical strategy trials.

## Data Availability

The datasets used and analysed during the current study are available from the corresponding author on reasonable request.

## Abbreviations

ADAb: Anti-drug antibody/-ies
Anti-CCP: Anti-cyclic citrullinated peptides
AS: Ankylosing spondylitis
ASDAS-CRP: Ankylosing Spondylitis Disease Activity Score-C-reactive protein
axSpA: Axial spondyloarthritis
bDMARDs: Biologic disease-modifying antirheumatic drugs
CI: Confidence interval
CII: Clinically important improvement
CZP: Certolizumab pegol
DAS28-ESR: 28-joint Disease Activity Score-erythrocyte sedimentation rate
DMARD: Disease-modifying antirheumatic drug
EULAR: European League Against Rheumatism
IQR: Interquartile range
IJD: Inflammatory joint diseases
OR: Odds ratio
PsA: Psoriatic arthritis
RA: Rheumatoid arthritis
RF: Rheumatoid factor
SD: Standard deviation
TNFi: Tumour necrosis factor alpha inhibitors
TDM: Therapeutic drug monitoring
